# Holocene Critical Zone dynamics in an Alpine catchment inferred from a speleothem multiproxy record: disentangling climate and human influences

**DOI:** 10.1038/s41598-019-53583-7

**Published:** 2019-11-28

**Authors:** Eleonora Regattieri, Giovanni Zanchetta, Ilaria Isola, Elena Zanella, Russell N. Drysdale, John C. Hellstrom, Andrea Zerboni, Luigi Dallai, Evdokia Tema, Luca Lanci, Emanuele Costa, Federico Magrì

**Affiliations:** 1 Dipartimento di Scienze della Terra, University of Pisa, Via S. Maria 53, 56126 Pisa, Italy; 2 Istituto di Geoscienze e Georisorse, IGG-CNR, Via Moruzzi 1, 56126 Pisa, Italy; 3 Istituto Nazionale di Geofisica e Vulcanologia INGV, Via della Faggiola 32, 56126 Pisa, Italy; 4 Dipartimento di Scienze della Terra, University of Torino, Via Valperga Caluso 35, 10125 Torino, Italy; 5 Department of Resource Management and Geography, University of Melbourne, Victoria, 3010 Australia; 6 EDYTEM, UMR CNRS 5204, Université de Savoie-Mont Blanc, 73376 Le Bourget du Lac cedex, France; 7 School of Earth Sciences, University of Melbourne, Victoria, 3010 Australia; 8 Dipartimento di Scienze delle Terra “A. Desio”, University of Milan, Via Mangiagalli 34, I-20133 Milano, Italy; 9 Dipartimento di Scienze Pure e Applicate, University of Urbino, Piazza della Repubblica 13, 61029 Urbino, Italy; 10 Associazione Gruppi Speleologici Piemontesi AGSP-Club Alpino Italiano, Torino, Italy

**Keywords:** Climate sciences, Environmental sciences, Solid Earth sciences

## Abstract

Disentangling the effects of climate and human impact on the long-term evolution of the Earth Critical Zone is crucial to understand the array of its potential responses to the ongoing Global Change. This task requires natural archives from which local information about soil and vegetation can be linked directly to climate parameters. Here we present a high-resolution, well-dated, speleothem multiproxy record from the SW Italian Alps, spanning the last ~10,000 years of the present interglacial (Holocene). We correlate magnetic properties and the carbon stable isotope ratio to soil stability and pedogenesis, whereas the oxygen isotope composition is interpreted as primarily related to precipitation amount, modulated at different timescales by changes in precipitation source and seasonality. During the 9.7-2.8 ka period, when anthropic pressure over the catchment was scarce, intervals of enhanced soil erosion are related to climate-driven vegetation contractions and occurred during drier periods. Immediately following the onset of the Iron Age (ca. 2.8 ka), by contrast, periods of enhanced soil erosion coincided with a wetter climate. We propose that the observed changes in the soil response to climate forcing were related to early anthropogenic manipulations of Earth’s surface, which made the ECZ more sensitive to climate oscillations.

## Introduction

**Figure 1:**
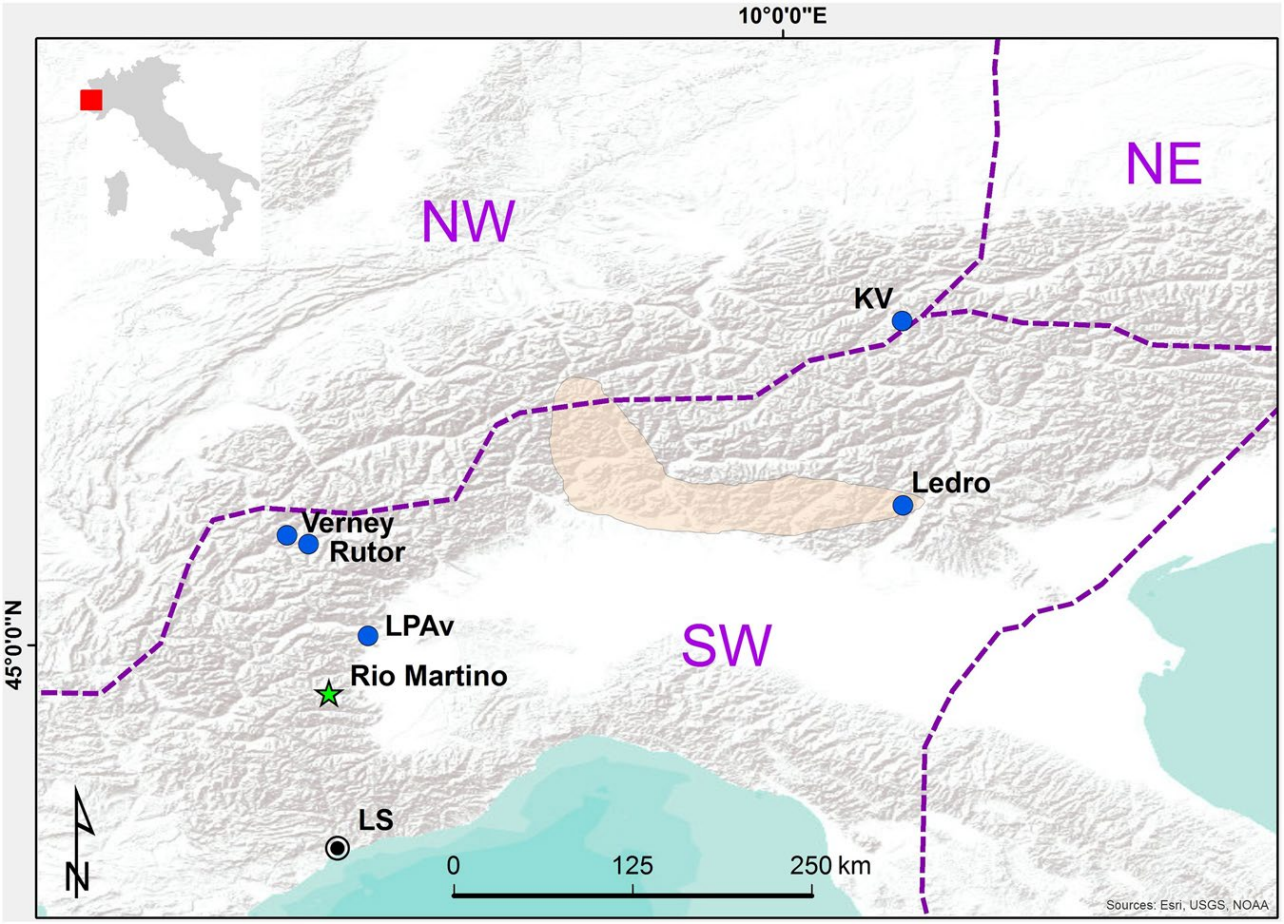
Location of Rio Martino Cave and of other sites mentioned in the text. Pink shadow indicates the area investigated in the synthesis on S Alps flood activity^30^; LS-Area of the cluster of deep-seated large scale landslides^45^; Ledro-Lake Ledro^29^; KV-Kauner Valley (tree-line elevation, Fig. 4);^53^ Rutor-Rutur mire (pollen percentage, Fig. 4)^41^; LPAv-Lake Piccolo di Avigliana (Urtica pollen %, Fig. 4)^36^ Vernet-Lake Verney^35^. Division of the Alpine region is from.

The Earth’s Critical Zone (ECZ) is the complex system of coupled physical, chemical, biological, and geological processes operating together to support life at the Earth’s surface. This dynamic interface extends from the vegetation top to the base of active groundwater, and is a constantly evolving envelope in which the soil represents a key component^1^. This zone is termed “critical” because it regulates the natural habitat and determines the availability of life-sustaining resources, but also because it is increasingly impacted upon human activities, especially land-use and land-cover change, which have long-lasting effects on the near-surface environment processes^2^. Natural ECZ functioning is regulated largely by climate through changes in temperature and hydrology, which impinge upon vegetation, soil, topography, weathering and erosion rates^2^. Understanding ECZ dynamics requires interdisciplinary approaches that span wide spatial and temporal scales^3^. Natural archives recording both soil processes and climate evolution are required to understand how soil development responds to climate variability and to human activity. Lake sediments and loess–paleosol sequences have intensively been studied in this context^4–6^. Speleothems (cave mineral deposits) are formed by meteoric water and receive inputs from bedrock, soil, vegetation and the atmosphere^7^. Geochemical properties of these deposits track environmental processes operating at the catchment scale^8^, as well as climatic changes acting at the regional scale in response to global or hemispheric climate patterns^9^. Although rarely exploited in this sense, speleothems are nevertheless well suited to reconstruct past soil dynamics and their relationship with climate, allowing us to disentangle the array of potential ECZ responses to climatic and land use changes. Here we present a high-resolution, multiproxy record (magnetic properties: natural magnetization intensity, *Jr*, mass magnetic susceptibility, χ, stable oxygen and carbon isotope composition: δ^18^O, δ^13^C and growth rate) obtained from a well-dated flowstone core (RMD1) from Rio Martino Cave (Piedmont, SW Italian Alps, Fig. 1), which grew continuously over the 9.7–0.4 ka interval, thus spanning most of the present interglacial^10^. Magnetic properties of RMD1 were investigated at high-resolution (~60 yr/sample), providing a record of regional paleosecular variations (PSV) of the Earth geomagnetic field during the Holocene^10^. Here we explore the paleoenvironmental significance of magnetic properties by comparing them to a high-resolution (~16 yr) stable isotope record (δ^18^O and δ^13^C) obtained from the same core. Magnetic minerals in speleothems are mostly related to detrital input transported by infiltrating waters^11^. The amount and distribution of the detrital component in speleothems are strongly dependent on geological, climatic, biological (including anthropic) processes governing soil formation^7^. The concentration, composition and grain size of the magnetic mineral assemblage may thus be used to reconstruct the pedological history at the catchment scale^11–14^. The stable isotope composition can provide information on paleo-precipitation, temperature patterns and/or soil-vegetation dynamics at the surface^7^, complementing the surficial processes recorded by the magnetic properties and linking local evolution to climate changes at the regional and extra-regional scale.

By coupling these different proxies, we attempt to reconstruct soil evolution at the catchment scale during the last ~10 kyr and its relation to local hydroclimate. We compare our reconstruction with the regional climatic and environmental framework, and with the record of human occupation, which is a pivotal factor in determining Holocene ECZ functioning, especially in the Alpine region^5^. The aim is to connect local processes in the soil and vegetation overlying the cave system to regional (Alpine) climatic changes and anthropogenic disturbance, to give insights on the links between the evolution of the ECZ, climate and human activities in the area during the Holocene.

### Settings and material

Rio Martino Cave opens at 1530 m a.s.l. in the upper Po Valley (SW Alps, Piedmont, northern Italy, Fig. 1). The catchment area lies between 1900 and 2200 m a.s.l. and is mantled by a thick (tens of meters) sedimentary cover comprising ophiolitic gravel with an abundant silty-sandy matrix, deposited and reworked by Quaternary glacial and periglacial processes. Currently, the catchment supports an alpine grassland dominated by C3 plants. The present natural timberline elevation for the southern Alps is about 2350 m^5^, thus the present vegetation is related to anthropic modifications. The cave is crossed by a permanent stream, which responds rapidly to meteoric events due to direct infiltration where the carbonate rock is closer to the surface. However, extreme discharge variations are dampened by the major contribution of slower water circulation occurring within the glacial cover, which represents the main aquifer feeding the river and the cave-drip recharge system^15^. A detailed description of the cave and of the regional climate is provided in SOM1.

RMD1 core is 59 cm long (Fig. SOM2-1) and shows predominantly columnar calcite (SOM2). Its age-depth model is based on 20 U/Th dates and has a mean uncertainty of 0.15 kyr^10^ (SOM3).

The main magnetic carrier, identified through Isothermal Remanent Magnetization (IRM) acquisition curves, is low-coercivity (saturation around 0.3 T) (titano)magnetite of detrital origin^10^. Hysteresis loops show that the magnetic grain-size is mostly in the single-domain (SD) to pseudo-single-domain (PSD) range. Some samples (mostly associated with *Jr* and χ spikes) also show the occurrence of magnetite in the multi-domain (MD) range^10^. The IRM is of detrital origin (DRM), i.e. acquired and locked-in soon after the calcium carbonate film deposition on the speleothem surface^11^.

### Results and paleoenvironmental significance of RMD1 proxies

**Figure 2:**
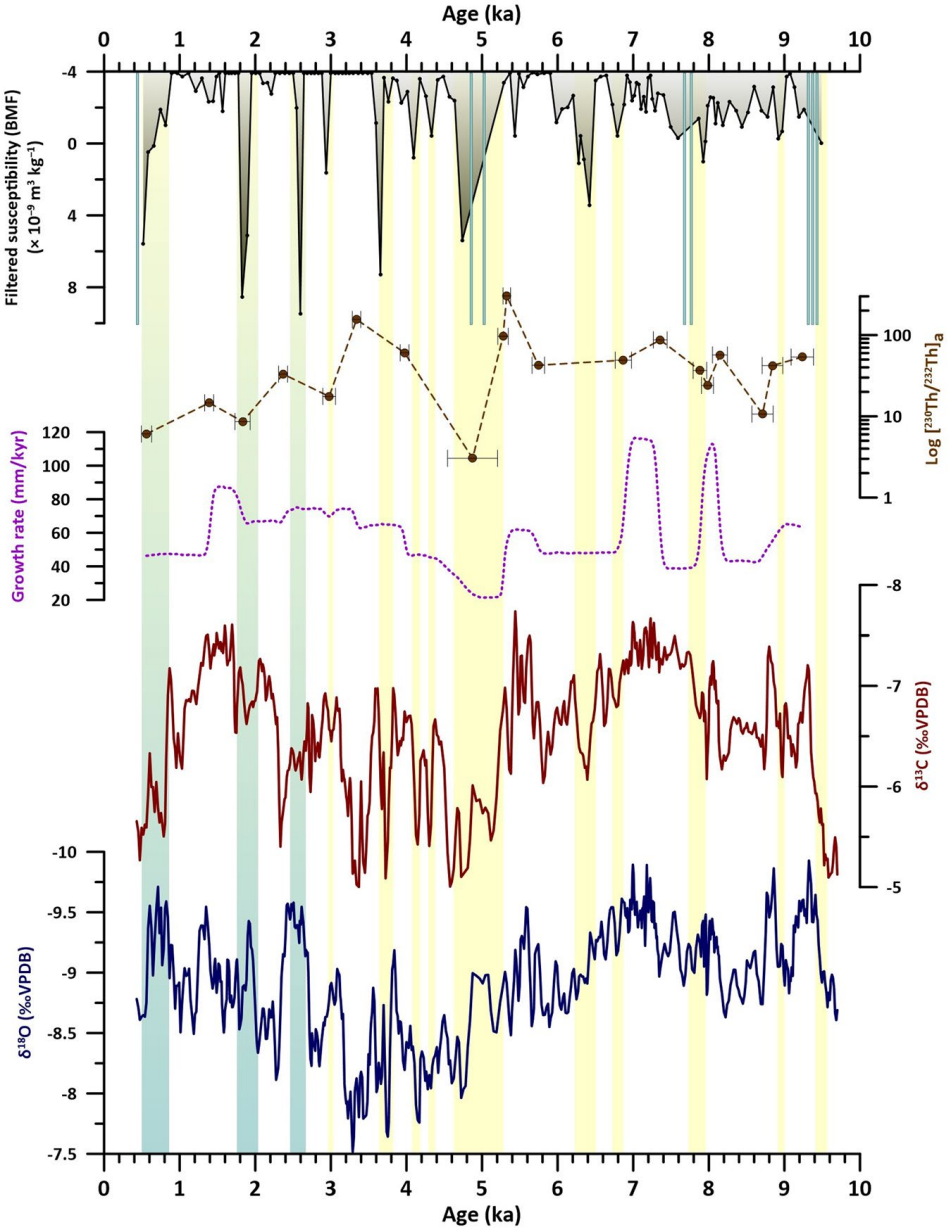
RMD1 proxies vs. age. From bottom: δ^18^O; δ^13^C, growth rate; [^230^Th/^232^Th] for the dated points; filtered mass magnetic susceptibility (χ) series representing the background magnetic flux (BMF, see text for details), light-blue bars are the χ peaks >12 m^3^ kg^−1^ indicating the spikes of coarser material. Rectangles indicate the spikes in BMF, yellow: spikes characterized by a similar behaviour of δ^13^C and δ^18^O; blue: intervals where δ^13^C and δ^18^O show an opposite pattern.

Assuming a constant diamagnetic contribution mostly related to the predominant calcite fraction, the relative variability of mass magnetic susceptibility (χ) represents variations in the concentration of magnetic minerals. A mean background value of −4 × 10^−9^ m^3^ kg^−1^ can be assumed for Rio Martino speleothem, while higher values correspond to the detrital input^10^. This is corroborated by the high correlation (r = 0.87) observed between χ and the natural magnetization intensity (*J*r)^10^. The χ depth-series of RMD1 consists mostly of a prevailing diamagnetic phase with small negative values, alternating with large positive spikes of up to 970 × 10^−9^ m^3^ kg^−1^ (Fig. SOM2-1). Major spikes are associated with coarser detrital grains containing small serpentinite lithics. Coarser detrital particles were likely deposited onto the flowstone by cave stream waters (as opposed to vadose drip waters) as part of their suspended load during flood events, or immediately after in the back-flooding phase^16^. In Rio Martino Cave, floods are associated with fast infiltration directly through the carbonate bedrock and occur mostly during episodic intense precipitation events and/or during snow-melt peaks in spring^15^. Aside from the major spikes, variations in the −4 × 10^−9^ m^3^ kg^−1^ to ~12 × 10^−9^ m^3^ kg^−1^ range, representing ~84% of χ data, can be assumed as representative of changes in the concentration of fine-grained material deposited via diffuse infiltration through the drip-feeding network. This fine-grained material is a flux of soil and regolith particles illuviated from the surface^16^. Soils of the Rio Martino catchment have developed on glacigenic ophiolitic sediments. Pedogenic, inorganic magnetite, formed by weathering of the iron-rich parent material, is the main component of the BMF signal (SOM4). At Rio Martino, this likely stems from the contribution of the “slow” component of the aquifer, which circulates in the glacial cover sediment. Thus, it can be used to infer changes in the background flux of material derived from the overlying soil and debris cover and delivered via drip water. To better highlight this subtle variability, we filtered the χ time series by removing the spikes related to the coarser fraction (i.e. above 12 × 10^−9^ m^3^ kg^−1^) and the values lower than −4 × 10^−9^ m^3^ kg^−1^, producing a time series of background magnetic flux (herein BMF, Fig. 2). The detrital origin of the BMF can be further tested by comparing it with the activity ratio between the radiogenic ^230^Th and the detrital ^232^Th (i.e. [^230^Th/2^32^Th]) of the dated samples, an independent proxy for detrital contamination^17^. Despite the very coarse temporal resolution, the [^230^Th/^232^Th] curve does show a similar pattern to the BMF series (Fig. 2). Changes in the detrital input can be related to changes in soil erosion rates and/or to changes in infiltration rates^13,14,18^.

**Figure 3:**
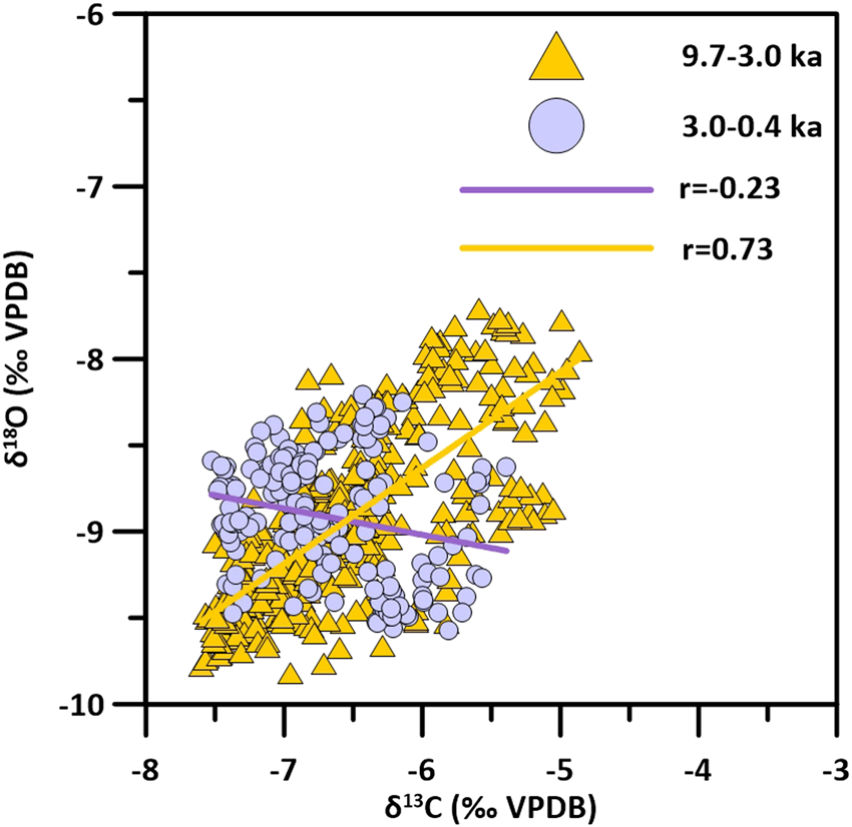
Correlations plot of δ^13^C and δ^18^O time series, previously smoothed with a 5-points running average.

Both stable isotope time series show similar multi-millennial trends from the base up to ca. 1.5 ka. Beyond this, the two series diverge, with decreasing δ^18^O values paired to strongly increasing δ^13^C (Fig. 2). Superimposed on this are several multi-centennial oscillations. From the base of the record up to 2.8 ka, oscillations are mostly in phase between the two isotope series. Conversely, from 2.8 ka the two centennial patterns appear anticorrelated, and prominent intervals of negative δ^18^O values are concomitant with δ^13^C increases (Fig. 2). Pearson *r* values between the two series, previously smoothed using a five-point running average, support the visual correlation (Fig. 3): in the interval from 9.7 to 3.0 ka there is a strong, statistically significant, positive correlation (r = 0.73; n = 427), which becomes weakly negative from 3.0 ka afterwards (r = −0.23, n = 164). At mid-latitudes, speleothem δ^13^C and growth rate are related to biological CO_2_ production, which is dependent from soil and vegetation conditions in the catchment^19,20^. Warmer and wetter periods usually enhance the production of biogenic, ^13^C-depleted CO_2_ and increases the growth rate^19,20^. Cold/dry conditions reduce the vegetation cover and the biogenic CO_2_ supply, and, in mountain settings such as Rio Martino, may enhance cryogenic processes, interrupting pedogenesis and promoting soil erosion. In these settings, an increase in the persistence of snow cover may also impact the δ^13^C by reducing the length of the plant growing season, leading to reduced biogenic CO_2_ supply. Lower/higher calcite δ^13^C values and faster/slower growth rates are therefore usually indicative of soil development/disruptions and can be related to warmer-wetter/colder-drier conditions. Additional control on the δ^13^C is exerted by the hydrological state of the aquifer. Partial dewatering of the drip-feeding system induces solution degassing and prior calcite precipitation, leading to higher δ^13^C during drier periods^7^.

**Figure 4:**
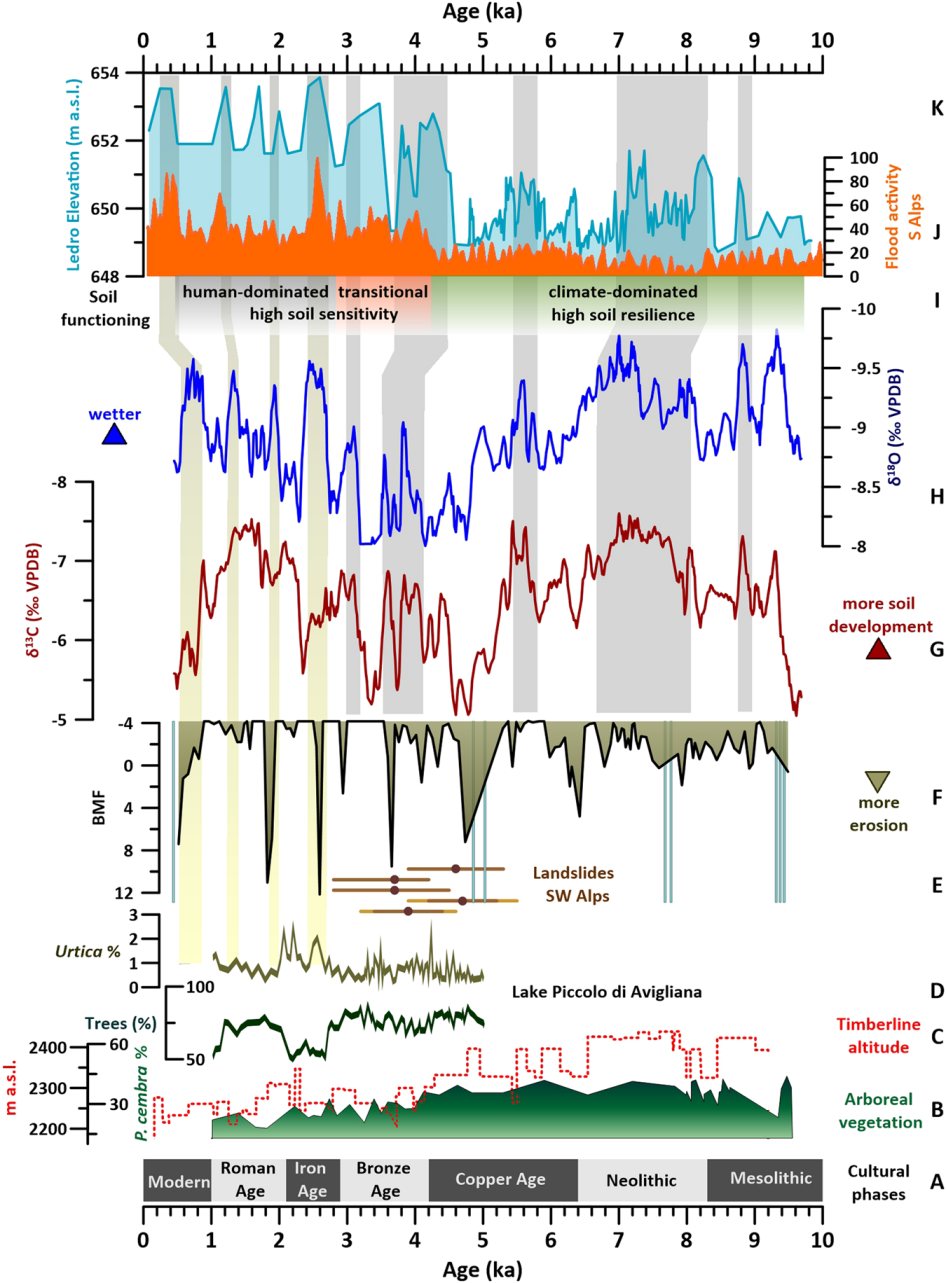
From bottom: A-Cultural phases in northern Italy^34^; B-*P. cembra* pollen percentages from Rutor Mire, SWAlps^41^; C-Alpine tree-line altitude from Kauner Valley^53^; D-pollen related to anthropic impact from Lago Piccolo di Avigliana^36^; E-ages of deep-seated landslides from SWAlps^45^; F-filtered magnetic susceptibility (BMF) from RMD1, light-blue bars indicate the peaks in coarser material; RMD1 δ^13^C (G) and δ^18^O (H), smoothed with a 5 points running average; I-Modes of soil functioning inferred from the RMD1 record J-Synthesis of flood activity from the S Alps^30^; K-Lake Ledro level variations^29^.

Paleoclimatic interpretations of speleothem δ^18^O mostly depend on cave location and setting^21^ (SOM5). In continental Europe and the Northern Alps, a positive temperature/δ^18^O relationship is usually reported^22^, whereas in the Mediterranean region a negative relationship is commonly observed between speleothem δ^18^O and the amount of precipitation^23,24^. Evidence for this latter “rainfall amount effect” has been reported for the Southern Alps^25,26^. Other drivers of alpine speleothem δ^18^O have been linked to shifts in precipitation seasonality and/or source^27,28^ (SOM5). For RMD1, several lines of reasoning (SOM5) suggest a first-order control of the amount effect on the δ^18^O record, although this is likely modulated at different time scales by changes in seasonality or in the source of precipitation. To test this hypothesis, we compared the δ^18^O with the record of lake-level variations from Lake Ledro^29^ and the synthesis of flood activity from the Southern Alps^30^ (Fig. 4). The observed similarities especially with the Lake Ledro record, confirm the proposed interpretation and suggest that the δ^18^O is representative of regional paleohydrology for the whole interval considered here, and thus provides a firm hydrological basis against which compare the proxies related to soil conditions (i.e. the BMF and the δ^13^C). Despite the good agreement between RMD1 δ^18^O and the Ledro record at the multi-centennial to millennial-scale (Fig. 4), the first part of the Holocene (i.e. up to ca. 5 ka) appears drier at Ledro whereas the RMD1 δ^18^O record shows relatively low values (i.e. wetter conditions). Changes in precipitation seasonality may explain the apparent discrepancy between drier conditions inferred from lake-level reconstructions and wetter conditions inferred from speleothem, because lower summer precipitation would increase the proportion of ^18^O-depleted winter precipitation, leading to more negative calcite δ^18^O values (SOM 5). A pollen-based quantitative reconstruction of winter and summer precipitation^31^ shows that extreme seasonality existed at Ledro only up to 8.5 ka, whereas the Mid- to Late Holocene was characterized by an increase in both summer and winter precipitation^31^. The most negative δ^18^O values observed in RMD1 occur prior to 8.5 ka and can be addressed to an additional influence of seasonality. On the contrary, the mismatching between 8.5 and 5 ka requires an alternative explanation. A pollen-based temperature reconstruction from the same study shows higher summer temperature at Ledro during the Early to Mid-Holocene^31^. This would cause higher lake-water evaporation rates and consequent lowering of the lake level, thus better explaining discrepancies between RMD1 δ^18^O and Lake Ledro in this interval. This is further supported by the occurrence of the lowest δ^13^C values in the first part (up to 5 ka) of the RMD1 record, because the δ^13^C is influenced by precipitation, but also by temperature, with warmer conditions enhancing soil biological activity^19^.

### Near-surface dynamics at Rio Martino: disentangling climatic and human influences

The comparison among the different RMD1 proxies shows that for the whole record most of the increases in BMF correspond to increases in δ^13^C and, in most cases, to a decrease in growth rate (Fig. 2). This is consistent with a close link between the soil status and vegetation on the one hand, and the detrital influx on the other, suggesting that the latter is dependent mostly on soil conditions. Between 9.7 and 2.8 ka, most of the increases in BMF and δ^13^C also correspond to increases in δ^18^O, indicating drier conditions (Fig. 2). This suggests that infiltration rates are not exerting a major control on the long-term pattern of soil erosion, whose increases appear related to periods of reduced precipitation and possibly cooler conditions, reducing the vegetation cover and enhancing the surficial erosion. Clearly, occasional heavy-rain events may have favoured the downward transport of detrital material, as suggested by the magnetic spikes, but they should have less impact on the slow-flow component of the aquifer through which the BMF is delivered to the flowstone.

Between 9.7 and 9.3 ka the RMD1 record shows increasing precipitation corresponding to a decrease in the BMF (Fig. 4), both indicative of post-glacial soil stabilization. The occurrence of intense precipitation events mobilizing coarser material, likely abundant in the catchment after the glacial retreat, is apparent (Fig. 4). Between 9.3 and 6.4 ka, the record shows the highest levels of precipitation and the maximum input of soil biogenic CO_2_. Intervals of reduced precipitation and possibly temperature occur at 9.2 ka and between 8.6 and 8.2 ka. These events show a timing agreement to the widely recorded climate anomalies at 9.2 ka^32^ and 8.2 ka^33^, but are not really prominent in the BMF record, which, within the 9.3–6.4 ka period, shows the highest background values and a muted variability (Fig. 3). The timberline for the western Alps was likely above 2350 m a.s.l. between 8.8 and 5.7 ka^34^. The background BMF over this period likely indicates a steady flux of material illuviated from a well-developed forest soil, where an increase in organic acids from roots and the litter boosted the mobility of Fe in the upper soil horizons and its translocation downward^35^. If we consider the anthropic influence, it is generally accepted that Mesolithic to Neolithic alpine hunter/gatherers caused only temporary ecosystem disturbances by fire intensification, rather than shaping ‘anthropogenic landscapes’^5,34,36^. Only sparse archaeological evidence is found for Neolithic exploitation of the high-altitude Alpine belt (i.e. above 2000 m)^37^, although alpine materials and artefacts were exchanged over great distances^37^. In the Rio Martino area, an extensive trade network likely already existed in the late Neolithic due to the importance of nearby Mont Viso as a source of greenstone axes^37^. However, human impact on the local ECZ dynamics should have been almost negligible (especially if compared with nearby lowlands), and the arboreal cover over the cave catchment would have limited soil erosion and the transport of detrital material, thus dampening the influence on soil of centennial-scale precipitation (and temperature) variability, as well as reducing the impact of extreme rain events.

Between 6.4 and 4.6 ka, the RMD1 record shows a trend of decreasing precipitation, reduced soil activity, and several multi-centennial increases in detrital influx. A prominent deterioration is apparent in all proxies between 6.4 and 6.0 ka (Fig. 4). Although not well expressed in the Lake Ledro record, it matches a dry/cold interval detected in several other Mediterranean terrestrial and marine records^38,39^. Also, between 5.2 and 4.6 ka, RMD1 shows another interval of higher soil erosion, corresponding to prominent drier conditions (Fig. 4). The pronounced lowering of growth rate (Fig. 3) suggests a temperature decrease. The maximum of this interval is also characterized by spikes in the coarser fraction, indicating an increase of floods and/or in the supply of erodible detrital material in the catchment, potentially related to a reduced arboreal cover. At the regional scale, the regime of soil formation from ~5.2 ka appears to change towards more unstable conditions and higher erosion rates^5^, whereas forest decline and lowering of the timberline, triggered by repeated phases of climate instability, are increasingly evident from 5.6 ka onwards^40,41^. In the Alps, the 6.4–4.6 ka interval also corresponds to the Early to Late Copper Age^34^. Sporadic occupation of high-altitude Alpine sites since this period is reported from archaeological remains and charcoal accumulation in lake sediments^5,40^. It has been suggested that climatic instability during the Mid-Holocene may have been a precursor to the development of high-altitude pastoralism rather than an obstacle for such activity due to increased pressures on lowland pasture and opened-up higher altitude pastures as tree-lines retreated^37^. However, persistent human impact on vegetation during the Copper Age was either absent or undetectable in mid- to high-altitude palynological records from both the northern and the Southern Alps^36,42^. Accordingly, at Rio Martino intervals of soil erosion appear to occur during drier conditions, and can be related to climate-driven vegetation contractions. From 4.6 ka, precipitation and soil conditions recovered at Rio Martino, but both remained highly variable at the multi-centennial scale until ~3.0 ka (Fig. 4), with abrupt and repeated drier intervals associated with a decrease in soil activity and increased detrital influx. At the regional scale, the period between ca. 5.5 and 3.0 ka corresponds to a substantial rearrangement of forest composition and further reduction in arboreal cover^34,41^, a widespread expansion of small alpine glaciers (the so-called Alpine Neoglacial period^43,44^), and an increased frequency of large-scale landslides in the SW Alps and the Apennines, which was related to periods of heavy rainfall^45,46^(Fig. 4). Over this time interval, there is much evidence for the onset of pervasive and diffuse anthropogenic, zoo-geomorphological disturbances (*sensu*^47,48^), with most records indicating that humans became a major geomorphological agent in shaping high-altitude Alpine environments after the Copper/Bronze Age transition (~4.2 ka, Fig. 4)^5,6,34,37,49^. In the surrounding Alpine foothills, increasing long-term trends in anthropogenic pollen reflect a more intensive human-impacted environment, because of a major change in agricultural practices related to the adoption of Bronze tools^36,42^. Contemporaneous dramatic changes in the engagement with the high-altitude Alpine zone are suggested by the appearance of stone-built structures in the SW Alps between ca. 4 and 3 ka^37^, which is consistent with the development of persistent summer settlements. Interestingly, from 3.9 ka onward, BMF/ δ^13^C spikes become more abrupt and shorter, and there is an overall lowering of the BMF background (Fig. 4). The onset of a human-induced “metapedogenetic phase”, characterized by definitive deforestation and erosion of the upper organic horizon and was identified between 4.3 and 2.6 ka in the record of Lake Verney from the French-Italian Alps^35^, located at 2188 m a.s.l, thus at an altitude comparable with Rio Martino. This is consistent with the higher sensitivity of soil conditions to hydrological variations apparent from our record since ca. 4.4–4.2 ka (Fig. 4), related to deforestation and increasing anthropic pressure over the catchment.

From 2.8 ka onward, the RMD1 carbon and oxygen time series largely decouple (Fig. 3). Intervals of depressed soil biological activity (higher δ^13^C) and enhanced detrital flux (higher BMF) appear to coincide with prominent intervals of lower δ^18^O, indicating wetter conditions. Negative peaks in the δ^18^O curve still resemble lake-level variations at Ledro (Fig. 4), and its general trend mimics the regional precipitation increase observed since 4.4 ka^5,29,50^. Thus, hydrology was still the first-order driver of δ^18^O, whereas there is a change in the δ^13^C response to hydrological variations. Following this, prominent intervals of abrupt increases in precipitation between 2.8 and 2.3 ka, at ca. 2.0 ka and between 1.0 and 0.5 ka are marked by deterioration of soil conditions, and higher erosion (Fig. 4). The pattern of variations in the different RMD1 proxies suggests a change in the soil response to climate forcing, and unprecedented environmental modifications initiated at ca. 2.8 ka. Noteworthy, this change corresponds to the beginning of the Iron Age in northern Italy^34^. At the Bronze to Iron Age transition (2.9–2.8 ka), increasing minerogenic input from soil overlaps with the widespread occurrence of anthropic indicators, such as *Urtica* in several lacustrine pollen records from both the northern and southern Alpine foothills^42^, and notably in the pollen record from Lake Piccolo di Avigliana^36^, not far from Rio Martino (Figs. 1 and 4). Progressively stronger impacts was related to pronounced demographic expansion, with reinforcement of herding at high-altitude sites paired with the spread of metal tools for agriculture and forest exploitation (Fig. 4)^5,34,40,49^. Interestingly, in the southern Alps the commencement of dairy production, especially that of hard cheese, is dated by some authors to the Late Bronze/Early Iron Age^51^. Milk cannot be stored for long periods and deteriorates rapidly, whereas cheese can be easily stored and transported. The development of dairy techniques allowed the use of upland summer pasture and the exploitation of the high-quality Alpine pastures for grazing, triggering the onset of seasonal transhumance and the rise of the modern-time “Alpine economy”^51^. This widespread land-use change, paired with climate-related regression of the timberline, would have had a dramatic impact on soil conditions. Therefore, we correlate changes in the relative pattern of hydrological variations and soil conditions at Rio Martino since 2.8 ka, -i.e. enhanced erosion occurring during wetter rather than drier periods- to the establishment of intensive grazing due to the definitive settlement of high-altitude pastoralism in the catchment. This triggered a rapid degradation and erosion of soil horizons, leading to an overall increase in the vulnerability of soils to climate change. In particular, it seems to have led to a marked enhancement of surface erosion and soil loss during periods when infiltration rates were high, likely resulting in a permanent shift from arboreal to the present-day shrub-and-grasslands vegetation.

In summary, the combined variability of magnetic parameters and the stable oxygen and carbon isotope composition from a Rio Martino speleothem provides new insights into the links between climate, soil stability and evolution, and land-use changes for the area during the Holocene, and has far-reaching implications for our understanding of the interplay between climate, human activities and ECZ dynamics. From 9.8 ka to 4.2 ka, when anthropic pressure at high-altitude Alpine sites was low^34–36^, intervals of soil instability and increasing erosion occurred during drier and possibly cooler periods, and were linked to climate-driven reductions in vegetation cover that are traceable at the regional scale. This influenced soil erosion and enhanced the detrital influx. Wetter and warmer periods, instead, promoted soil stability, with the denser vegetation retaining micrometer-scale pedogenic minerals and reducing detrital flux to the cave. Between 4.2 and 2.8 ka, intervals of high erosion were shorter, more frequent and abrupt, but still coinciding with drier periods, suggesting that soil sensitivity was enhanced by deforestation during the Bronze Age. From 2.8 ka onwards, with the beginning of the Iron Age, the onset of seasonal transhumance and the establishment of the modern “Alpine economy”^51^, increased exploitation of high-altitude sites resulted in a drastic change in the response of soil stability to climate variations, with intervals of soil erosion promoted by increasing precipitation. This suggests that early land-use changes made the mountain pedosphere more sensitive to climatic oscillations, and that pre-industrial human impacts profoundly modified the natural functioning of the ECZ in this Alpine catchment, causing heterogeneities in its responses to climate forcing. Our findings fit well within the recent global synthesis of land-use for the last 10 ka which reveals a planet Earth well and truly transformed by growing communities of hunter-gatherers, farmers, and pastoralists by 3 ka^52^. The RMD1 record also demonstrates that speleothem paleomagnetic properties are an effective tool to reconstruct past soil dynamics and their link with natural and anthropic processes, potentially advancing our ability to understand the present and future evolution of the ECZ and its potential responses to ongoing climate and land-uses changes.

## Methods

Details of the U/Th dating, age modelling and paleomagnetic analyses have been reported previously^10^, and are summarized in SOM3. Samples for stable isotope analysis were drilled on a polished half of the core at 1 mm increments using a milling lathe (CNC Protosystem) at the National Institute of Geophysics and Volcanology (Pisa). Stable oxygen (δ^18^O) and carbon (δ^13^C) isotope analyses on 495 subsamples were performed with a Gas Bench II (Thermo Scientific) coupled to a Delta XP (Finnigan MAT) IRMS at the Institute of Geosciences and Earth Resources of the Italian National Research Council (IGG-CNR, Pisa). About 0.12 mg of calcite were digested in H_3_PO_4_ (105%) and left to react at 70 °C for 1 h. Sample results were corrected using the IAEA standard NBS-18 and a set of three internal standards, which were calibrated by inter-laboratory comparisons and using the IAEA standards NBS-18 and NBS-19 and. Results are referred to the V-PDB international standard. Analytical uncertainties are of 0.15‰ and 0.10‰ and for δ^18^O and δ^13^C respectively.

## Supplementary information

Supplementary Information 

Supplementary information
