# Supplementary material for: Holocene Critical Zone dynamics in an Alpine catchment inferred from a speleothem multiproxy record: disentangling climate and human influences
Source: Sci Rep. 2019 Nov 28;9:17829. doi: 10.1038/s41598-019-53583-7 (PMC6883060; doi:10.1038/s41598-019-53583-7)
Supplement: Supplementary file 1 — Supplementary Information [file 41598_2019_53583_MOESM1_ESM.docx]

**Supplementary Online Material**

**Holocene Critical Zone dynamics in an Alpine catchment inferred from a speleothem multiproxy record: disentangling climate and human influences**

by E. Regattieri et al.

**SOM 1- Site settings**

*SOM 1.1 Cave Description*

Rio Martino Cave is a spring cave (3421 m of total length, 191 m of height difference) composed by two main sub-horizontal branches separated by a waterfall of 40 m in height. It is carved within the Mesozoic dolomitic carbonate cover of the Palaeozoic Dora Maira metamorphic unit (Balestro et al., 2013). The carbonate body is almost not outcropping in the catchment because it is covered by a tens of meters-thick cover of Quaternary sediments of glacial and periglacial origin and mostly deriving from disruption of the Monviso meta-ophiolitic complex overlying the carbonates (Magrì, 2007).

Mean cave temperature, assessed during a three-year monitoring program (2004-2006) is 5.3 °C (Magrì et al., 2007). Mean annual precipitation is ca. 1100 mm, with maxima in April-May and October-December. The gridded map of δ^18^O composition of precipitation in Italy (Giustini et al., 2016) reports values between -12‰ and -10‰ at the cave location. Mean annual temperature from the Crissolo meteorological station is 7.9°C, ranging from ca. -15°C (January) to 26°C (August) (data from ARPA Piemonte, http://www.arpa.piemonte.it/).

*SOM 1.2 Regional climatic framework*

Spatial patterns of long-term mean precipitation and temperature in the Alpine Region are the result of the combined influence of several large-scale climatic regimes prevailing over the Atlantic, the European continent, and the Mediterranean Basin (Wanner et al., 1997). Alpine climate is modulated by topography and by complex multi-scale interactions between the mountain range and a variety of weather and flow systems (Frei and Schär, 1998). The most prominent effects of alpine topography include the enhancement of precipitation along the foothills and rain shadows over the interior valleys (Frei and Schär, 1998), as well as the presence of a distinctive temperature field for regions located at altitudes higher that 1000 m a.s.l. (Efthymiadis et al., 2007). Precipitation over the Southern Alps is significantly correlated to that of southern Europe, particularly with the Western Mediterranean (Efthymiadis et al., 2007). The North Atlantic Oscillation (NAO) is the main large-scale atmospheric mode affecting temperature and precipitation in the southern Alpine region (Efthymiadis et al., 2007). The NAO index, i.e. the difference between normalized pressure series for Gibraltar and SW Iceland (e.g. Jones et al., 1997), shows statistically significant positive correlations with temperatures. For precipitation, there is a more marked spatial and temporal variability, but on average there is a significant negative correlation between NAO index and winter precipitation, which is stronger for the Mediterranean sectors of the alpine chain (Lopez-Moreno et al., 2011), and at higher altitudes (Casty et al., 2005). This is due to the fact that negative/positive NAO indices causes a southern/northern shift of westerly circulation, allowing a greater/reduced penetration of moisture-bearing air masses from the North Atlantic to the Mediterranean. Negative/positive NAO phases also stimulate/depress cyclogenesis within the basin, particularly that occurring in the Gulf of Genoa (Reale and Lionello, 2014), the most active cyclogenetic centre in the Mediterranean Region. Precipitation in the Southwest Alps appears to be sensitive also to a Scandinavian-centered atmospheric pressure pattern (Wibig, 1999), with an inverse correlation between western Scandinavian and southern Alps barometric-pressure patterns. These findings mostly relate to the winter months, when the dynamical relationship between European (and of the extratropical Northern Hemisphere, in general) surface climate and large-scale atmospheric circulation is most pronounced. Since precipitation is not uniformly distributed during the year, the overall influence of the principal atmospheric circulation patterns on the annual average has been quantified at ca. 45% for the southwestern sectors of the Alpine chain (Efthymiadis et al., 2007). During summer, European pressure gradients are weaker and the influence of large-scale circulation patterns is less important, while local and/or subregional conditions (mostly due to thermal effects) become more influential (Efthymiadis et al., 2007).

*SOM 1 References*

Balestro, G., Fioraso, G., Lombardo, B. Geological map of the Monviso massif (Western Alps*). J. Maps* **9** (4), 623–634 (2013).

Magrì, F. and Associazione Gruppi Speleologici Piemontesi (AGSP). La Grotta di Rio Martino (Valle Po-Piemonte). Eds. Federico Magrì. Pubs. Regione Piemonte, 5-98, (2007).

Casty, C., *et al.* Temperature and precipitation variability in the European Alps since 1500. *Int. J. Clim*., **25**(14), 1855-1880 (2005).

Efthymiadis, D. et al. Influence of large-scale atmospheric circulation on climate variability in the Greater Alpine Region of Europe, *J. Geophys. Res.,* **112**, D12104 (2007),

Frei, C., Schär, C.A. Precipitation climatology of the Alps from high‐resolution rain‐gauge observations. *Int. J. Clim.,* **18**(8), 873-900. (1998).

Giustini, F., Brilli, M., & Patera, A. Mapping oxygen stable isotopes of precipitation in Italy. *Journal of Hydrology: Regional Studies*, **8**, 162-181 (2016).

Jones, P. D., Jonsson, T., Wheeler, D. Extension to the North Atlantic Oscillation using early instrumental pressure observations from Gibraltar and south‐west Iceland. *Int. J. Clim* **17**(13), 1433-1450 (1997).

López-Moreno, J. I. *et al.* Effects of the North Atlantic Oscillation (NAO) on combined temperature and precipitation winter modes in the Mediterranean mountains: Observed relationships and projections for the 21st century. *Glob. Pla. Change* **77**(1), 62-76 (2011).

Reale, M., Lionello, P. Synoptic climatology of winter intense precipitation events along the Mediterranean coasts. *Natural Hazards and Earth System Science*s, **13**(7), 1707-1722 (2013).

Wanner, H., *et al.* Global climate change and variability and its influence on alpine climate—concepts and observations. *Theoretical and Applied Climatology*, **58**(3-4), 221-243 (1997).

Wibig, J. Precipitation in Europe in relation to circulation patterns at the 500 hPa level. *Int. J. Clim.*, **19**(3), 253-269 (1999).

**SOM 2- The core RMD1**


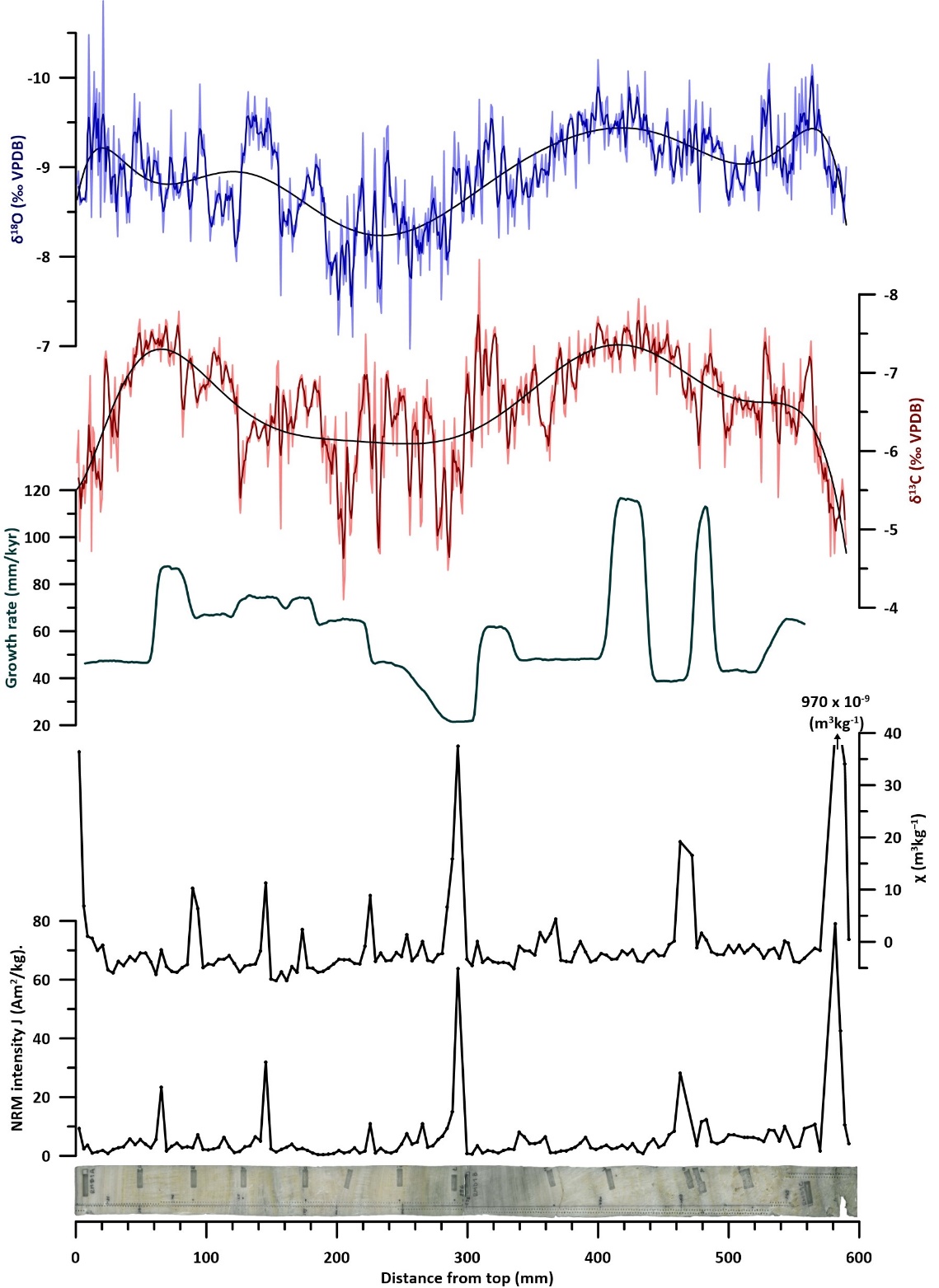


Figure SOM2-1 The RMD1 core and depth series of RMD1 proxies.

*SOM2-2 Petrographic analyses*

Three thin sections were cut for petrographic analyses, at 550-590 mm, 330-290, 60-20 mm depths from top (section Bottom-Middle-Top; Fig. S3). The thin sections were observed using a transmitted-light microscope (Zeiss Laboval 4 ausJena) and photographed using a digital camera (Canon EOS).

Thin sections analyses show predominantly columnar calcite, grading from micro-crystalline sub-type in the basal portion (~12 cm from the bottom) to compact and elongated columnar18 in the upper part. The columnar fabric in speleothem is considered indicative of near-equilibrium calcite precipitation, with reduced influence of random kinetic isotope fractionation (Frisia and Borsato, 2010).

The preservation of the paleosecular record demonstrates that diagenetic processes, such as dissolution and recrystallization, do not affect the speleothem. The mineralogy of the acid-resistant <0.45 μm fraction is in strong agreement with catchment lithology, being mostly composed of soil-derived iron oxides, magnesium silicates and serpentine-group minerals sourced from the glacigenic sediment cover (Zanella et al., 2018).


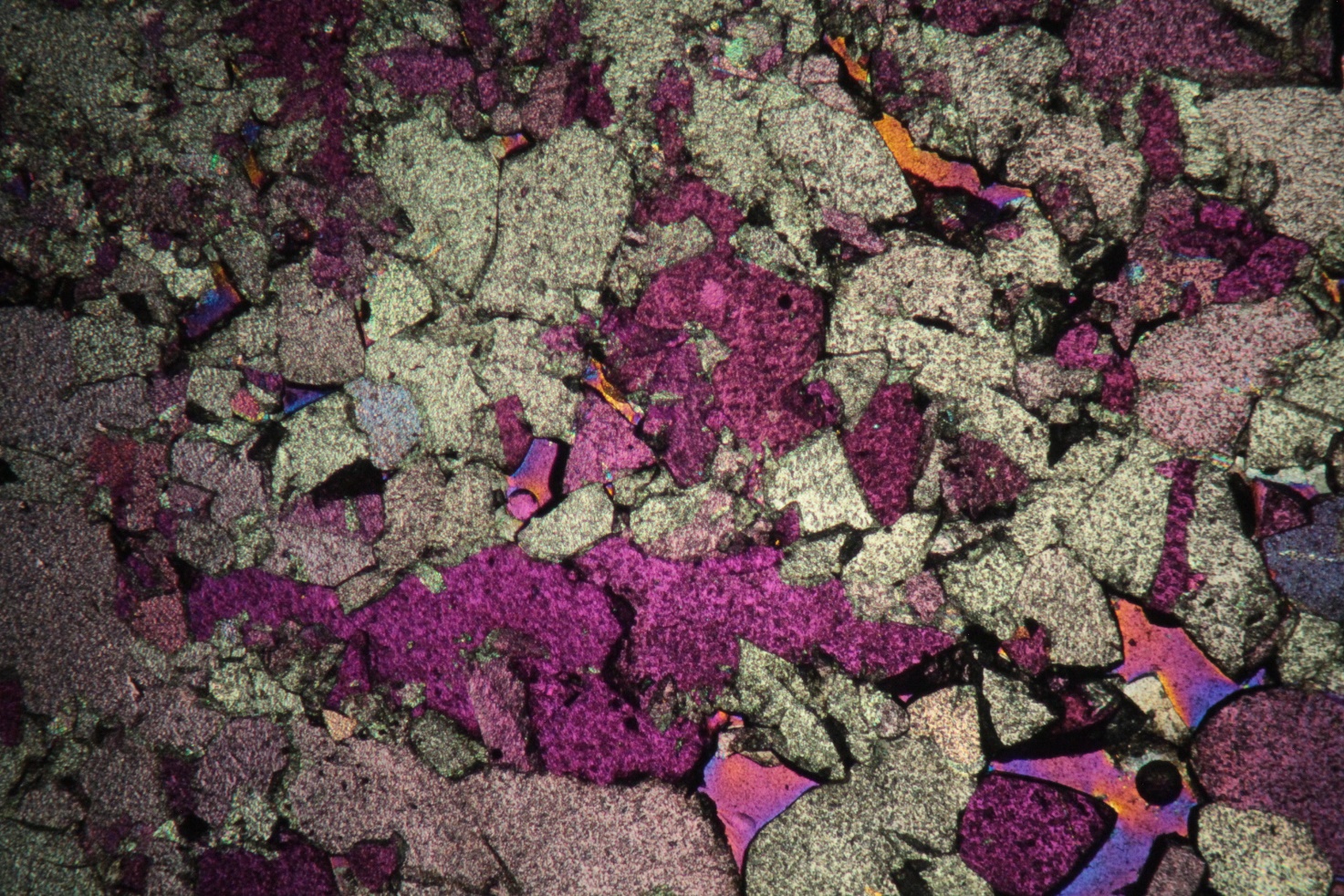


Figure SOM2-2 Microcristalline columnar calcite from section RMD1 bottom (crossed nicols, 2.5X, base of the photo 5.2 mm)


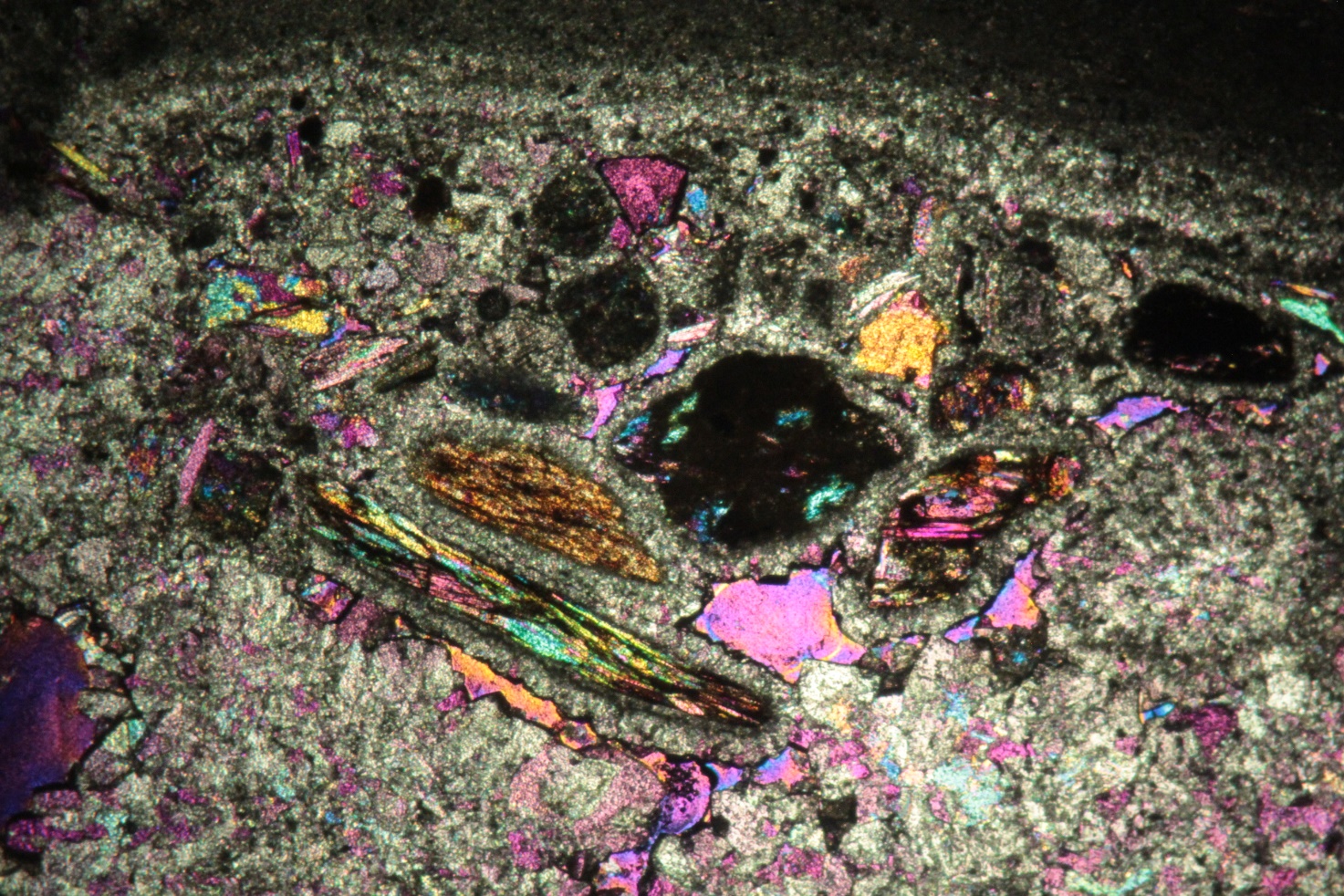


Figure SOM2-2 Coarser detrital particles at the base of section RMD1 bottom, corresponding to the first susceptibility spike (crossed nicols, 2.5X, base of the photo 5.2 mm).


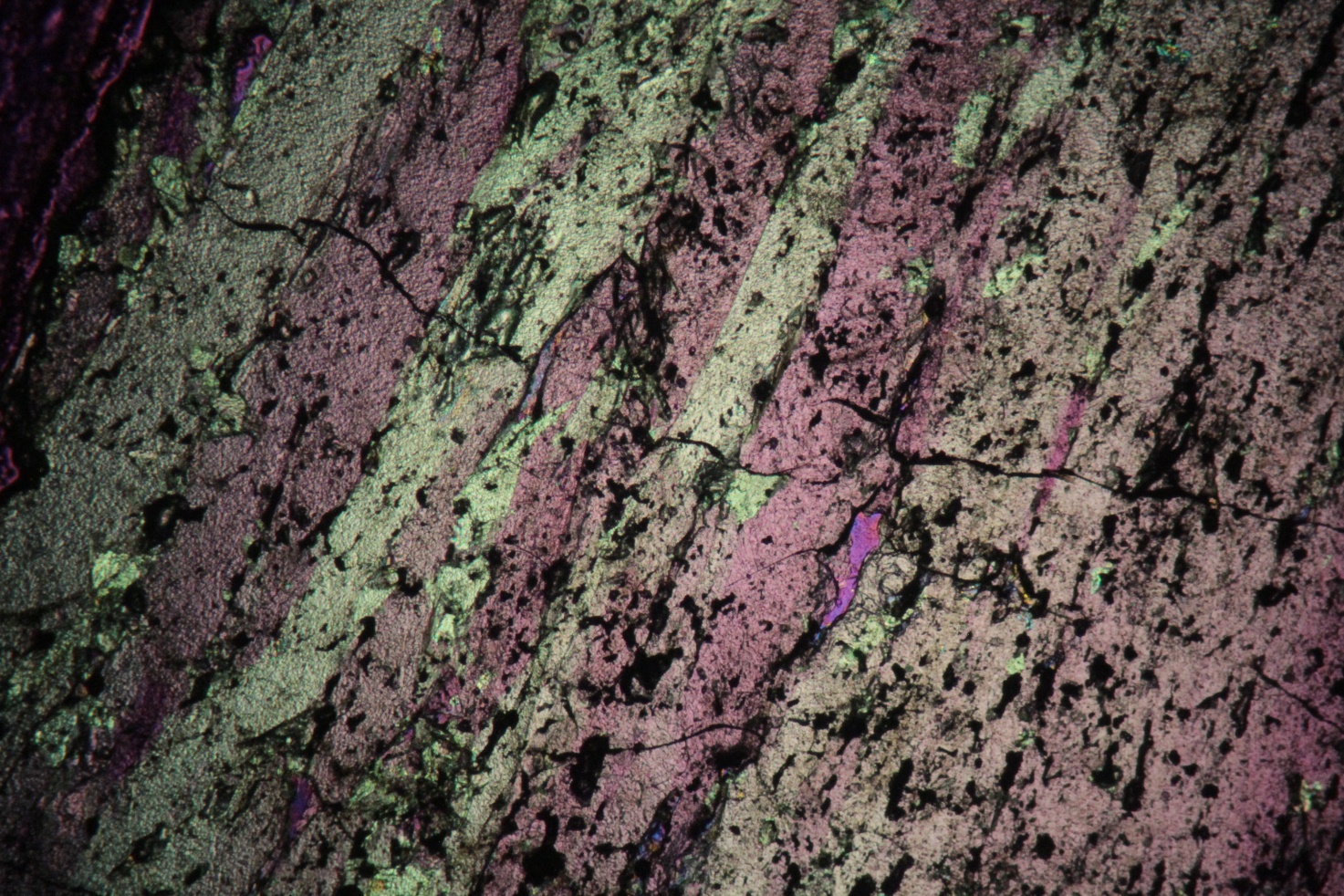


Figure SOM2-2 Elongated columnar calcite from section RMD1 top (crossed nicols, 2.5X, base of the photo 5.2 mm).

SOM 2 References

Frisia, S., Borsato, A. Karst. In Carbonates in continental settings. *Development in Sedimentology* **61**, 269–318. (2010).

Zanella, E., *et al.* A 10,000 yr record of high-resolution Paleosecular Variation from a flowstone of Rio Martino Cave, Northwestern Alps, Italy. *Earth Planet. Sc. Lett.* **485**, 32-42 (2018).

**SOM 3- Methods for previously published data**

Details of U-Th dating and age-depth modelling on the RMD1 flowstone, as well as paleomagnetic measurements, are provided in Zanella et al. (2018). A brief summary of the methods and results is provided here.

*SOM 3-1 U/Th dating and age modelling*

Nineteen solid prisms of ∼40 mg (∼2 mm wide along the lamina and 1 mm thick on growth axis) from RMD1 were used for age determination (Table S1). The U/Th dating was performed at the University of Melbourne (Australia) following the method of Hellstrom (2003). Samples were dissolved and (^236^U–^233^U–^229^Th) spiked and the carbonate matrix was removed. The purified U and Th fraction was introduced to a multi-collector inductively coupled plasma mass spectrometer (MC-ICPMS, Nu-Instruments Plasma). The ^230^Th/^238^U and ^234^U/^238^U activity ratios were calculated from the measured atomic ratios and calibrated against the HU-1 secular equilibrium standard. Correction for detrital Th content was applied using an initial activity ratio of detrital thorium [^230^Th/^232^Th] of 1.3 ±0.45. This value, and its relative 2σ uncertainty, was calculated following Hellstrom, (2006) using a Monte Carlo stratigraphic-constraint procedure based on the U/Th ages. The depth–age model (Fig. S1) presented here is based on the U/Th ages reported in Table S1. It was constructed employing a Bayesian Monte Carlo approach (Drysdale et al., 2005). The resulting isotope time series covers the interval between 0.41 ± 0.12 ka and 9.7 ± 0.39 ka.

| Sample ID | ^238^U (ng/g) | Depth (mm) | ^(230^Th/^238^U)*10^3^ | **^234^U/^238^U** | **Age uncr. Ka** | (^232^Th/^238^U)*10^3^ | ^230^Th/^232^Th | **Age cr Ka** | 2se (kyr) |
| --- | --- | --- | --- | --- | --- | --- | --- | --- | --- |
| RMD1-A | 579 | 8 | 8.2 | 1.26 | 0.707 | 1.353 | 6.1 | **0.553** | 0.043 |
| RMD1 137.5 | 885 | 50 | 18.0 | 1.29 | 1.530 | 1.229 | 14.7 | **1.396** | 0.046 |
| RMD1 130.4 | 898 | 90 | 25.2 | 1.29 | 2.140 | 2.922 | 8.6 | **1.825** | 0.074 |
| RMD1 126 | 773 | 128 | 28.8 | 1.29 | 2.453 | 0.870 | 33.1 | **2.366** | 0.048 |
| RMD1 128.6 | 1124 | 176 | 37.7 | 1.30 | 3.196 | 2.149 | 17.5 | **2.970** | 0.068 |
| RMD1 135.6 | 726 | 204 | 39.4 | 1.30 | 3.344 | 0.253 | 155.3 | **3.328** | 0.053 |
| RMD1 133.5 | 1097 | 248 | 47.8 | 1.31 | 4.047 | 0.792 | 60.4 | **3.969** | 0.040 |
| *RMD1 139.6** | *683* | *289* | *81.5* | 1.26 | *6.667* | *26.757* | *3.0* | ***4.220*** | *0.694* |
| RMD1-B | 1135 | 299 | 62.2 | 1.28 | 7.281 | 0.642 | 96.9 | **5.308** | 0.058 |
| RMD1-301 | 971 | 301 | 61.3 | 1.28 | 5.358 | 0.202 | 302.8 | **5.331** | 0.039 |
| RMD1-328 | 887 | 328 | 67.5 | 1.28 | 5.334 | 1.588 | 42.5 | **5.728** | 0.069 |
| *RMD1 157.4*** | *926* | *368* | *86.0* | *1.28* | 5.889 | *3.029* | *28.4* | ***7.225*** | *0.084* |
| RMD1-386 | 925 | 386 | 80.5 | 1.28 | 7.033 | 1.638 | 49.2 | **6.875** | 0.093 |
| RMD1-445 | 894 | 445 | 84.0 | 1.27 | 7.415 | 0.964 | 87.1 | **7.331** | 0.072 |
| *RMD1 136.7*** | *1055* | *462* | *76.3* | *1.28* | 8.194 | *0.571* | *133.6* | ***6.622*** | *0.059* |
| RMD1 173.3 | 559 | 467 | 92.4 | 1.27 | *7.539* | 2.521 | 36.7 | **7.931** | 0.093 |
| RMD1 107.1 | 437 | 478 | 92.5 | 1.24 | 8.400 | 3.852 | 24.0 | **7.977** | 0.114 |
| RMD1-497 | 915 | 497 | 93.9 | 1.28 | 8.262 | 1.651 | 56.9 | **8.101** | 0.086 |
| RMD1 M5.6 | 490 | 523 | 110.7 | 1.26 | 9.971 | 10.362 | 10.7 | **8.824** | 0.298 |
| RMD1-530 | 573 | 530 | 102.6 | 1.28 | 9.084 | 2.437 | 42.1 | **8.838** | 0.151 |
| RMD1-C | 600 | 557 | 106.7 | 1.28 | 9.391 | 1.974 | 54.0 | **9.202** | 0.140 |

Table S1- Corrected (in bold) and uncorrected U/Th ages for RMD1 flowstone core and related 2σ uncertainties expressed in ka before 2010. The activity ratios have been standardized to the HU-1 secular equilibrium standard, and ages calculated using decay constants of 2.835 × 10^−6^ for ^234^U and of 9.195 ×10^−6^ for ^230^Th. Depths are mm from the core top. Ages with asterisk were rejected as outliers.


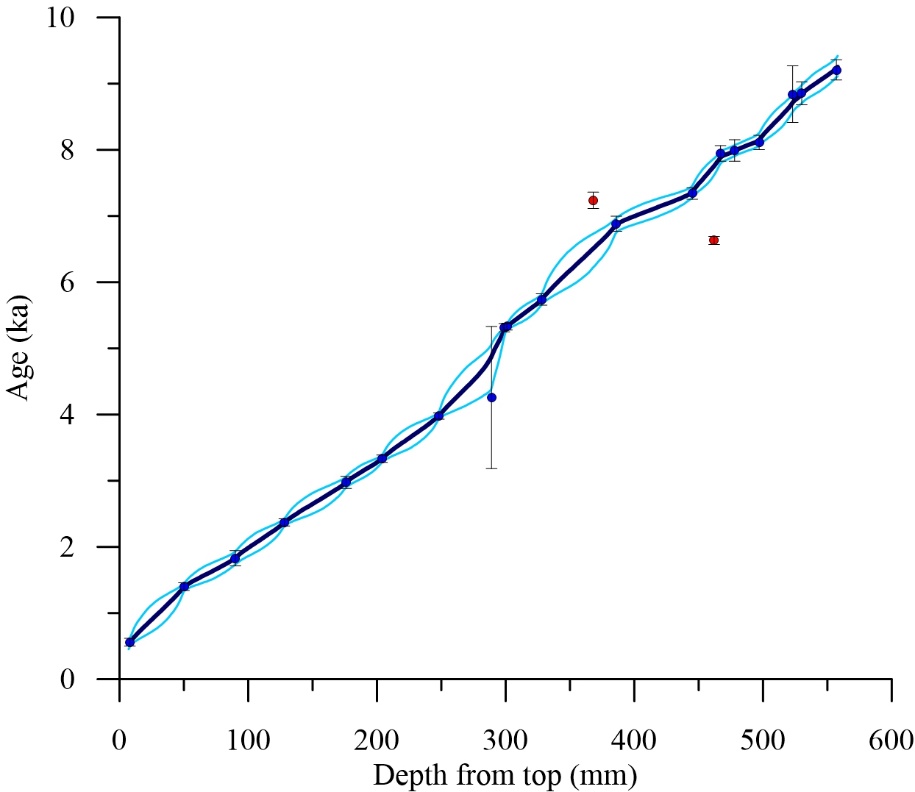


Figure S2-Age-depth model for core RMD1. Light-blue lines represent the 95% confidence interval. Ages rejected as outlier are shown in red.

*SOM 3-2 Magnetic measurements*

Magnetic measurements were performed on 146 thin speleothem slices using a non-magnetic plastic holder specifically designed for this study at the CIMaN-ALP Laboratory (Peveragno, Italy). For each specimen, the magnetic susceptibility (k_m_) and natural remanent magnetization (J_r_) were measured by a k-Bridge (KLY-3) and a spin magnetometer (JR6) (both housed at AGICO), respectively. Slices were weighed to obtain their mass-values.

After having Af step by step demagnetized up to 100 mT each specimen, an Anhysteretic Remanent Magnetization (ARM) was imparted using a 100 mT AF field with a 0.15 mT direct current (DC) biasing field. Then IRM acquisition and Lowrie’s experiment (Lowrie, 1990) were performed on selected specimens. ARM and IRM data were used to test the occurrence of magnetic interaction and to assess the magnetic grain-size, as described by the method of Symons and Cioppa (2000) and Cisowski (1981). Magnetic mineralogy results are given in Zanella et al. (2018).

*SOM 3 References*

Cisowski, S. Interacting vs. non-interacting single domain behavior in natural and synthetic samples. *Physics of the Earth and Planetary Interiors* **26**, 56-62 (1981)

Drysdale, R.N., *et al*. Stalagmite evidence for the onset of the Last Interglacial in southern Europe at 129 ± 1 ka. *Geophysical Research Letters* **32**, 1–4 (2005).

Hellstrom, J.C., Rapid and accurate U/Th dating using parallel ion-counting multicollector ICP-MS. *Journal of Analytical Atomic Spectrometry* **18**, 1346–1351 (2003).

Hellstrom, J.C. U-Th dating of speleothems with high initial 230Th using stratigraphical constraint. *Quat. Geochronology* **1**, 289–295 (2006)

Lowrie, W., Identification of ferromagnetic minerals in a rock by coercivity and unblocking temperature properties. *Geophysical Research Letters* **17**, 159–162 (1990).

Symons, D.T.A., Cioppa, M.T. Crossover Plots: a useful method for plotting SIRM data in paleomagnetism. *Geophysical Research Letters* **27**, 1779–1782 (2000)

Zanella, E. *et al.* A 10,000 yr record of high-resolution Paleosecular Variation from a flowstone of Rio Martino Cave, Northwestern Alps, Italy. *Earth Planet. Sc. Lett*. **485**, 32-42 (2018).

**SOM 4 Origin and significance of RMD1 paleomagnetic signal**

As for Rio Martino, most of the research dealing with speleothem magnetic properties has identified fine-grained magnetite derived from the soil as the main magnetic carrier (Lascu and Feinberg, 2011; Bourne et al., 2015; Jaqueto et al., 2016; Zhu et al., 2017). Magnetite in soil could either be of detrital or authigenic origin. Detrital magnetite is produced from erosion of igneous and metamorphic rock. It is characterized by larger grain sizes, mostly MD, with Fe often substituted by Ti, V, Cr (Taylor et al., 1986). Authigenic (i.e. formed in the soil as consequence of pedogenesis) magnetite is characterized by smaller grain sizes (PSD to SD). It derives from precipitation of Fe^3+^ produced by oxidation at the mineral surface. This process is favored in well-drained, low-acid soils (pH ~5.5-7), developing on weatherable, Fe-bearing parent materials (Taylor et al., 1986; Maher and Taylor, 1988). Bacterial biomineralization of magnetite is also an important mechanism of magnetic enhancement in soil and leads to scarcely substituted, ultrafine-grained magnetite (Taylor et al., 1986; Maher and Taylor, 1988). Due to the high presence of magnetic minerals in the ophiolitic glaci-genic cover of Rio Martino catchment we assume a reduced relative importance of bio-mineralization

This is supported by the predominant SD-to-PSD range observed from RMD1 samples (Zanella et al., 2018). Interestingly, the presence of MD, higher-coercivity magnetite derived from erosion of the ophiolitic bedrock occurs in correspondence of the high-magnetic spikes (Zanella et al., 2018), suggesting it is mostly related to the mobilization of the coarser fraction by the quick-flow component of the aquifer

*SOM 4 References*

Bourne, M. D. *et al.* Long-term changes in precipitation recorded by magnetic minerals in speleothems. *Geology* **43**(7), 595-598 (2015).

Jaqueto, P*. et al.* Linking speleothem and soil magnetism in the Pau d'Alho cave (central South America). *J. Geophys. Res.-Solid Earth* **121**(10), 7024-7039, (2016).

Lascu, I. & Feinberg, J. M. Speleothem magnetism. *Quat. Sci. Rev.* **30**(23-24), 3306-3320 (2011).

Maher, B. A. & Taylor, R. M. Formation of ultrafine-grained magnetite in soils. *Nature* **336**(6197), 368. (1988).

Taylor, R. M., Maher, B. A. Self, P. G. Magnetite in soils: I. The synthesis of single-domain and superparamagnetic magnetite. *Clay min.* **22**(4), 411-422 (1986).

Zanella, E., *et al.* A 10,000 yr record of high-resolution Paleosecular Variation from a flowstone of Rio Martino Cave, Northwestern Alps, Italy. *Earth Planet. Sc. Lett.* **485**, 32-42 (2018).

Zhu, Z., *et al.* H. Holocene ENSO-related cyclic storms recorded by magnetic minerals in speleothems of central China. *Proc. Nat. Acad. Sci. U.S.A.,* **114**(5) 852-857 (2017).

**SOM 5 Paleoclimatic interpretation of speleothem δ^18^O composition**

Oxygen stable isotope composition of calcite precipitating close to equilibrium conditions depends on the temperature and δ^18^O composition of the precipitating solution (Epstein et al., 1953). A negative temperature dependence is observed, and a fractionation coefficient of -0.177‰°C^−1^ has been empirically derived for speleothem calcite (Tremaine et al., 2011), and recently updated to 0.2‰°C^−1^, a value which incorporates uncertainties related to kinetic fractionation (Daëron et al., 2019). The δ^18^O of drip water in relatively deep caves from temperate to arid settings mostly represents the weighted mean annual δ^18^O value of precipitation (e.g. Genty et al., 2014). The δ^18^O of the precipitation is positively correlated with atmospheric temperatures and negatively correlated with rainfall amount (Dansgaard, 1964). The strength of these correlations varies depending on geographic setting, resulting in site to site variations in the drivers of speleothem calcite δ^18^O composition. In the Mediterranean Basin, the rainfall δ^18^O-to-temperature gradient is ~ +0.2‰ (Bard et al., 2002), thus counterbalancing the temperature-dependent isotope fractionation (~-0.2‰) during carbonate precipitation. Conversely, the modern relation between rainfall δ^18^O and precipitation amount is highly negative, ~ -1.6‰ per 100 mm/month (Bard et al., 2002), leading to lower/higher speleothem δ^18^O values associated with wetter/drier conditions. In northern and central Europe the rainfall δ^18^O-temperature relationship is strongly positive (0.58‰/°C, Rozansky et al., 1993). Once the carbonate temperature-dependent isotope fractionation is subtracted, this results in a positive correlation between speleothem δ^18^O and temperature (0.58‰ - 0.2 ‰= 0.38‰°C^-1^), and thus to more negative speleothem δ^18^O values associated with lower temperatures, and *vice versa* (e.g. Boch et al., 2011). Northern alpine speleothems typically show, as well, a positive relationship between δ^18^O and temperatures (Holzkamper et al., 2004; Boch et al., 2011; Hauselmann et al., 2015). Instead, from southern Alps evidences for a predominant amount effect as drivers for speleothem δ^18^O variations have been reported (Belli et al., 2013; Columbu et al., 2018). Other drivers proposed for Alpine speleothem δ^18^O are related to changes of ^18^O-depleted winter vs. ^18^O-enriched summer effective infiltration ratio (Mangini et al., 2005; Vollweiller et al., 2006), and to shifts in the main precipitation source (i.e. the Atlantic and the Mediterranean, Lüetscher et al., 2015). To add complexity, the influence of the local topography and the development of local thermal low-pressure centres, triggering cyclogenesis to occur locally, can dominate the local temperature and precipitation patterns for large part of the year (Trigo et al., 2002), strongly imprinting the mean annual δ^18^O values of the precipitation at the specific cave location. This means that often it is not straightforward to disentangle a predominant climatic driver for the alpine δ^18^O signal, which is subjected to multiple and contrasting influences. As a fact, clear common trends can be hardly identified between different alpine speleothem δ^18^O records, nor strong relationships with the regional framework inferred from other archives such as lakes and pollen sequences (e.g. Magny et al., 2009, Furlanetto et al., 2018) can be firmly established, especially at the centennial-millennial scale.

For RMD1 several lines of reasoning suggest a first-order control of the amount effect on the δ^18^O record, though likely modulated at different time scales by changes in seasonality or in the source of precipitation. First, the general trend and most of the centennial-scale variability show a common pattern with the δ^13^C (notwithstanding the decoupling observed in the last ~3 ka of the record, to be addressed below), with intervals of more negative δ^18^O corresponding to warm and/or wet conditions inferred from the carbon record (Fig. 2). On the contrary, a negative covariance would be expected if temperature is controlling the δ^18^O. Second, the interval of highest δ^18^O is broadly coincident with the major growth rate reduction indicating lower temperature and/or drier conditions (Fig. 2). Finally, due to the relative proximity (ca. 100 ka) of Rio Martino site to the northern coast of the Genoa Gulf (where the amount effect was defined, Bard et al., 2002), synoptic conditions occurring in this area likely contribute to precipitation patterns at the cave site, causing a stronger Mediterranean character for this sector of the Alps (i.e. a stronger influence of the amount effect on δ^18^O of the precipitation). On a longer-time perspectives in fact, the pollen-based reconstruction of Mauri et al. (2015) shows that winter precipitation in the Southern Alps were significantly correlated to that of the Mediterranean during the whole 12-0 ka period.

*SOM 5 References*

Bard, E., *et al*.. Hydrological conditions over the western Mediterranean basin during the deposition of the cold Sapropel 6 (ca. 175 kyr BP). *Earth Planet. Sc. Lett.*  **202**, 481–494 (2002)

Belli *et al.* Regional climate variability and ecosystem responses to the last deglaciation in the northern hemisphere from stable isotope data and calcite fabrics in two northern Adriatic stalagmites. *Quat. Sci. Rev*, **72**, 146-158 (2013)

Boch, R., *et al.* NALPS: a precisely dated European climate record 120–60 ka, *Clim. Past.,* **7**, 1247–1259 (2011)

Columbu, A. *et al.* Palaeoenvironmental changes recorded by speleothems of the southern Alps (Piani Eterni, Belluno, Italy) during four interglacial to glacial climate transitions. *Quat. Sci. Rev.* **197**, 319-335 (2018).

Dansgaard, W. Stable isotopes in precipitation. *Tellus*, **16**(4), 436-468 (1964).

Daëron, M. *et al.* Most Earth-surface calcites precipitate out of isotopic equilibrium. *Nat. Commun*., **10**(1), 429-432 (2019).

Furlanetto, G. *et al.* Holocene vegetation history and quantitative climate reconstructions in a high-elevation oceanic district of the Italian Alps. Evidence for a middle to late Holocene precipitation increase. *Quat. Sci. Rev.* **200**, 212-236 (2018)

Genty, D.*et al.* Rainfall and cave water isotopic relationships in two South-France sites. *Geochim. Cosmochim. Acta*, **131**, 323-343 (2014).

Häuselmann, et al. Timing and nature of the penultimate deglaciation in a high alpine stalagmite from Switzerland. *Quat. Sci. Rev.*, **126**, 264-275. (2015).

Holzkämper, S. *et al.* Timing and progression of the Last Interglacial derived from a high alpine stalagmite. *Geophysical Research Letters*, **31**(7). (2004).

Lüetscher, M. *et al.* North Atlantic storm track changes during the Last Glacial Maximum recorded by Alpine speleothems. *Nat. Commun.* **6**, 6344 (2015).

Magny, M., *et al.* Holocene palaeohydrological changes in the northern Mediterranean borderlands as reflected by the lake-level record of Lake Ledro, northeastern Italy. *Quat. Res.* **77**(3), 382-396 (2012).

Mangini, A., Spötl, C., Verdes, P. Reconstruction of temperature in the Central Alps during the past 2000 yr from a δ^18^O stalagmite record. *Earth Planet. Sc. Lett.* **235**(3-4), 741-751 (2005).

Mauri, A *et al.* The climate of Europe during the Holocene: a gridded pollen-based reconstruction and its multi-proxy evaluation. *Quat. Sci. Rev*., 112, 109-127 (2015).

Rozanski, K., Sonntag, C., Münnich, K. O. Factors controlling stable isotope composition of European precipitation. *Tellus*, **34**(2), 142-150 (1982).

Tremaine, D.M., Froelich, P.N., Wang, Y., Speleothem calcite farmed in situ: modern calibration of δ^18^O and δ^13^C paleoclimate proxies in a continuously-monitored natural cave system. *Geochim. Cosmochim. Acta* **75** (17), 4929–4950 (2011).

Trigo, I. F., Bigg, G. R., Davies, T. D. Climatology of cyclogenesis mechanisms in the Mediterranean. *Monthly Weather Review*, **130**(3), 549-569 (2002).

Vollweiler, N. *et al.* (2006). A precisely dated climate record for the last 9 kyr from three high alpine stalagmites, Spannagel Cave, Austria. *Geophys. Res. Lett*., **33**(20).
